# Application of Elastic Waves and Neural Networks for the Prediction of Forces in Bolts of Flange Connections Subjected to Static Tension Tests

**DOI:** 10.3390/ma13163607

**Published:** 2020-08-14

**Authors:** Piotr Nazarko, Leonard Ziemiański

**Affiliations:** Department of Structural Mechanics, Faculty of Civil and Environmental Engineering and Architecture, Rzeszow University of Technology, Powstancow Warszawy 12, 35-959 Rzeszow, Poland; ziele@prz.edu.pl

**Keywords:** elastic waves, signal processing, neural networks, force prediction, flange connection, static test

## Abstract

There is a group of measurement techniques that can be used in the task of force identification in steel bolts. In this paper, the potential of elastic wave propagation signals was studied for possible application in force monitoring systems. A series of laboratory tests was carried out on flange connections subjected to static tensile tests. Each one contained six screws of the same diameter. Four bolts were equipped with washer load cells. Alternatively, selected bolts were equipped with piezoelectric transducers (actuator and sensor) in order to measure the elastic wave signals. Principal components analysis, time of arrival, and neural network compression were used for dimensionality reduction of the measured signals. Examples of the obtained results with respect to the studied connections show that the tension forces in bolts can be estimated with relatively good accuracy.

## 1. Introduction

Measurements of the physical quantities that describe structural elements’ state are significant in many engineering and industrial applications, including civil engineering. They are often conducted pending trial loads of new structures and prototype solutions in laboratory tests. This allows a better understanding of the behavior of the entire structure and its individual elements. Non-destructive tests (NDT) and structural health monitoring systems (SHM) are very useful in this task. Their constant use also increases the safety and reliability of the structure. There are also several tasks that can be carried out with their help [1,2,3,4,5,6]:

- Anomaly or damage detection;
- Damage type classification;
- Load or internal force prediction;
- Material parameter identification.

There is a group of structure connections where the pretension force level has an influence on the strength of the slip resistance connection. Changing pretension forces, i.e., due to the structure’s usage over time, may become a highly relevant matter for the structural integrity. It is especially important in cyclically loaded constructions like telecommunication towers, bridges, and wind turbines.

Besides the necessity to monitor the state of strategic structures, the precision of the tightening method implemented to accomplish an accurate preload level is equally important. One of the hazards related to the occurrence of failure or structural damage may be the insufficient stress ratio of screw connections. Among the methods used to control their tightening level, two main features can be distinguished: accuracy and practical applicability. Determining the prestressing force in everyday applications is most often based on the tightening torque measured with a dynamometer wrench. The accuracy of this approach is defined as ±25%. The best accuracy here is accomplished by using ultrasonic sensing (±1%) or strain gauges. There are already a few commercial solutions that can be used to estimate the axial forces in bolts. One of them concerns ultrasonic load-monitoring devices, which most often require access to bolts in a joint from both sides. The second solution is related to piezoelectric load cells or washer-type strain. The measurement uncertainty in this case amounts to ±10%. Fric et al. compared some of these methods [7]. However, their costs cannot be ignored, and therefore, in long-term research, strain gauges are used, either fitted inside the bolt shank or glued onto the bolt. For this reason, the article presents the results of laboratory research aimed at the use of commonly available and relatively inexpensive piezoelectric transducers (PZT) that can be used to excite and receive elastic waves.

In this work, the elastic wave propagation phenomenon was implemented due to the possibility of adjusting their parameters to the applied task, their sensitivity, and their ease of implementation in SHM systems. To measure and introduce the time signals of elastic waves, piezoelectric transducers were used. Nazarko and Ziemiański [8] used this idea also in the field of non-destructive damage detection in a variety of elements and materials. Chaki and Bource [9] examined guided ultrasonic waves to monitor and observe the stress levels in steel strands. In these approaches, including ultrasonic testing, the most frequently used parameters are changes in longitudinal and transverse wave propagation velocities or time of flight/arrival (ToF/ToA). In the discussed problem of identifying axial forces in bolts of flange connections, this turned out to be insufficient. Therefore, in order to obtain reliable signal parameters, their deep compression was applied. Additionally, as the inference tool, artificial neural networks (ANNs) were used [10].

The results presented in the article concern a series of laboratory tests carried out on flange connections under tension in a static test machine. It appeared that force changes have an impact on the signals measured by the sensors [11]. This was reflected also in the calculated principal components, which are often used to compress signals (see Nazarko [12] or Chen et al. [13], where other methods concerning signal compression were compared). This article presents new results where ANNs were used both for signal compression and as an inference tool. The value of these results is increased by the fact that they refer to experimental studies, where there are a number of factors (measurement noise, apparatus limitations) that are quite difficult to examine in numerical simulations and analytical calculations. In this case, the research concerned the possibility of predicting axial forces in tension bolts of flange connections. The elastic waves were regularly excited and recorded (by PZT sensors) during the connection tensile test. After extending the set of patterns (compressed signal parameters) with information about the elongation of one of the bolts, it became possible to estimate (using ANNs) the magnitude of axial forces not only within a single connection, but also to generalize the results within several analyzed connections. Moreover, these preliminary results show that ANNs are able to predict the axial forces in bolts with reasonably good accuracy. This also indicates the significant potential of the approach being developed for real-life NDT inspections.

## 2. Materials and Methods

### 2.1. The Idea of Force Monitoring in Bolts

The idea of bolt force monitoring discussed in this paper is based on measurements of elastic waves. It was confirmed by Kim and Hong [14] and Ding et al. [15] that even a relatively small change in a bolt force will affect the signals measured (its time of flight, amplitude, frequency, etc.). Changes in the tension force have an influence on the shortening/elongation of the screw. This results in variations occurring in the distance traveled by waves and the time related to it. Thus, the propagation velocity and ToF are the parameters frequently used to determine and detect the stresses or forces of the monitored elements [16].

**Figure 1:**
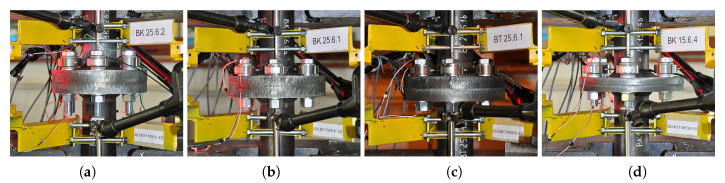
Example of connections subjected to static tests and the elastic wave propagation measurements in bolts: (**a**) P1, (**b**) P2, (**c**) P3, (**d**) P4.

In this paper, two single PZT were implemented to excite and to measure the response signals in the bolts of the studied flange connections (Figure 1). These transducers were placed in pitch–catch configurations. At the beginning, a single screw was equipped with additional transducers (one on the end of the shank and two on the screw’s head) in order to get a pulse–echo configuration. Nevertheless, due to the fact that both of them provided similar accuracy considering force identification, it was decided to use pitch–catch in subsequent studies because this enables one to obtain a higher signal-to-noise ratio (SNR).

Taking into account the simplicity of conducting on-site inspections, the most convenient placement of measurement units would be probably an impulse–echo configuration, but this was not considered in this study. In the case of SHM systems, whether or not the structure is equipped with instrumentation during its construction can be seen as irrelevant (actuators and sensors can even be mounted inside the bolts).

**Figure 2:**
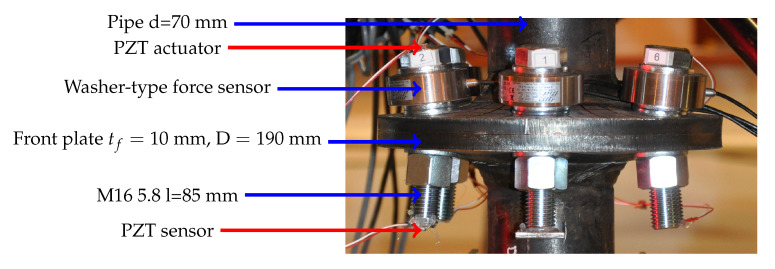
An example of visible bolts deformation at the end of the static test (P6 connection).

A typical washer force sensor consists of three or four standard piezoelectric measuring elements or strain gauges inside the unit. This is to average the results over the entire sensor surface. This approach should be considered in future work related to the approach under consideration because of the possibility of the simultaneous tensioning and bending of bolts (Figure 2).

Parameters describing the force changes (e.g., physically measured or determined based on the measured signals) can be used for training an efficient diagnosis system that is based on ANNs. The advantage of this over existing solutions is that the application of the proposed approach may allow one to estimate not only changes in forces over time, but also detect yielding and fatigue damages and monitor the structural integrity.

### 2.2. Laboratory Setup

The laboratory setup consisted of a signal generator (40 MHz DDS Function/Arbitrary Generator, TG4001, Thurlby Thandar Instruments Ltd., United Kingdom), in which an excitation was defined in the form of a 2.5 sine wave modulated by a Hanning window. The operational frequency was set to 52 kHz. Then, the signal was amplified and split into actuators and synchronization channels. Two digital oscilloscopes (WaveSurfer 424 and WaveRunner 104MXi, LeCroy, NY, USA) were used to store the signals received from all the sensors. Piezoelectric transducers (CMAP6, plate 5×5×2 mm, Noliac) were mounted on the bolt heads (excitation) and at the ends of their shanks (response). Sensor wax was used to attach them (this enabled trouble-free recovery of all the sensors), while their cables were fixed to a single spot with a weak adhesive to hold them during the test, allowing for non-invasive removal.

As usual, the measuring system used (piezoelectric transducer, amplifier, signal generator) has its limitations. This applies to both the excitation frequency and the signal amplification. This is one of the most important factors that affects the obtained accuracy of identification. In this case, the frequency and gain were selected in such a way that the response signal had the highest SNR at the highest possible frequency.

**Figure 3:**
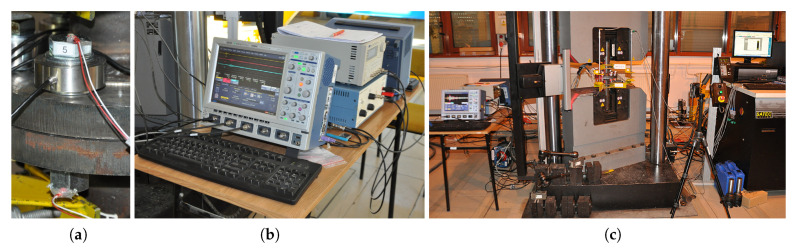
Laboratory setup: (**a**) Piezoelectric transducers mounted at the bolt ends (a washer-type force sensor is also visible in this case). (**b**) Digital oscilloscopes, function generator, and amplifier. (**c**) Static test machine Instron J1D 1200 kN.

A set of static tests was carried out using the testing machine Instron J1D 1200 kN (Figure 3). To evenly load the oscilloscopes that store the measurement data (saving data at the same time), it was assumed that each one would register the pattern of the excitation signal (in order to synchronize the recorded signals in time) and the response from the two screws.

### 2.3. Flange Connections under Static Tests

**Figure 4:**
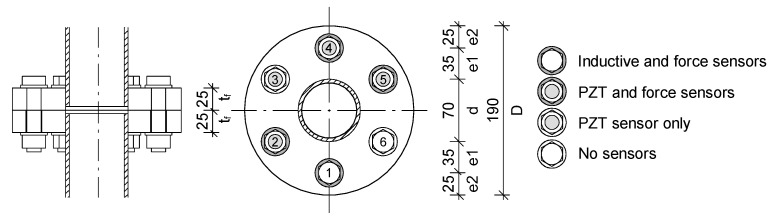
A scheme of the flange connection (P1/P2), screw numbers, and sensor locations (all dimensions are given in mm; washer-type sensors are not included).

The investigated set of flange connections consisted of six M16 bolts (Figure 4). In each connection, only four of them were equipped with washer strain load cells (Bolt Nos. 1, 2, 4, and 5; see Figure 4). The other two had spacers that imitated the force sensors in order to ensure the similar nature of bolt work. The dimensions of the considered connections and the types of screws are listed in the Table 1.

Unfortunately, as a result of technical complications (a lack of free hard disk space during the measurements) and the resulting differences in the number of recorded signals between the two oscilloscopes, measurement data from the P5* connection were not used in this article.

Two inductive sensors were mounted on screw S1 (from above and below; see Figure 1) to measure its displacement. Thanks to this, it was possible to determine the elongation of this screw during the tensile test. The other two inductive sensors were attached on the right and left side of the pipe rod. They were used to monitor possible rotational deformation, which was considered insignificantly small. On this basis, it was assumed that the investigated flange connections generally deform symmetrically, and the elongation of a single bolt can be used as an additional parameter describing their current condition. The conducted tests did not take into account the occurrence of a complex stress state, in which this assumption could be false.

**Figure 5:**
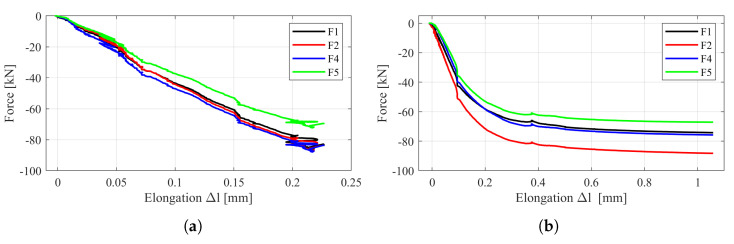
Elongation and forces measured in the bolts: (**a**) P1 connection; (**b**) P2 connection (see Table 1).

The forces obtained during the static tests and the elongation in one representative screw were used to define an output vector for ANN training. The obtained relationship between these values in the case of single connections is shown in Figure 5. It can be seen there that the forces measured in the bolts were not exactly the same. This may be related to the geometric imperfections of the connection (non-symmetric holes) or measurement uncertainty (this type of applied force sensor is sensitive to the washer hardness or a screw touching the edge of the hole).

In addition, four bolts were equipped with piezoelectric transducers, but their numbers were different with respect to the force sensor locations, i.e., 2, 3, 4, and 5 (Figure 4). It was not possible to mount them at the S1 bolt because the inductive sensors were placed there. As a consequence, the signals measured in the S3 bolts (in all connections), which were not equipped with the washer load cell, were used to define the database with unknown force magnitudes. After the process of ANN training, these data were used to predict the forces in those bolts.

It is worth mentioning here that the completed test plan did not assume cyclical loading/unloading of the connections. In this particular case, each individual test was intended to cause damage in one of the following forms: bolts yielding or breaking, plastic deformation of pipes or head plates. The failure model depended on the connection dimensions and the class of bolts used. For example, in the P1 connection, there was plastic deformation of the pipe observed (Figure 5a), while in the case of the P2 connection (see Figure 5b), the deformation of the pipes was accompanied by yielding of the bolts. This explains the differences that can be seen in these charts. Despite this fact, an attempt was made to estimate the bolt forces regardless of the connection operation phase.

In the experimental studies carried out, it was not possible to obtain measurement data from the unloading phase of a damaged joint in the case of all considered models. When the load capacity of the connection was being approached, some of the sensors were disassembled due to the risk of their damage. Therefore, it was decided not to include these data in the ANN training process. However, the issue of cyclic loading is very important, and further research in this area is needed. Examples of experimental studies carried out so far and the preliminary results of the axial forces’ identification can be found in the literature [11,17].

### 2.4. Signal Analysis

**Figure 6:**
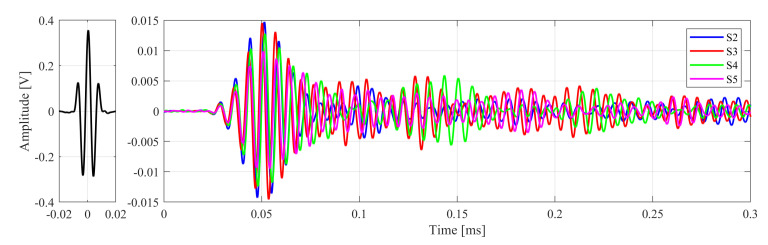
Excitation signal and the received responses measured at the S2 to S5 bolts in the P1 connection.

The elastic wave signal comparison in the initial stage showed that they were not exactly the same (Figure 6). There may be several reasons for this: the piezoelectric transducers may not have been positioned precisely in the same place (the central axis of the bolt); the thickness of the wax layer was not the same; the signals were affected by the closest components of the connection (washers, brackets on the pipe, weld thickness, etc.). Despite these differences, an attempt was made to train the ANNs for the purpose of force prediction. It was even decided to perform signal normalization within the range ±0.9 (with respect to the initial stage), but this did not significantly affect the results of the predicted force values.

As a result of the length of each signal, it was necessary to determine the parameters sensitive to load changes in the connections. Thus, the measured signals were transformed into the domain of the principal components [8,12]. This allows one to compute the linear transformation:

(1) y=W·s

which maps data from a high-dimensional space $s\subset R_{N}$ to a lower dimensional space $y\subset R_{K}$ of the principal components, without much loss of information. In this way, the elastic wave signals, each containing 10,002 points, after decimation at the rate r=2, were reduced to only 12 principal components.

**Figure 7:**
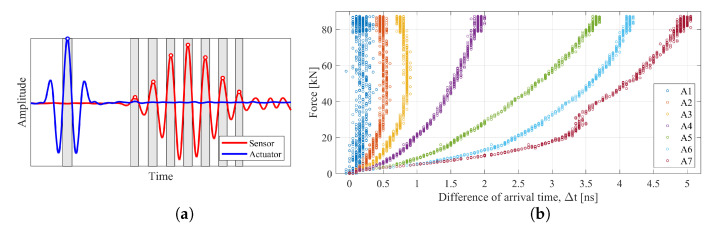
Arrival time and amplitudes: (**a**) an example of how they were determined; (**b**) differences of arrival time in the P1 connection.

On the basis of the preliminary test results [11], it turns out, however, that the principal components alone may not be sufficient for proper training of the diagnosis system. The point is for the ANNs to acquire generalization capabilities that enable the prediction of force values in bolts in which no control measurements were carried out. In other words, the obtained values of the estimated forces in the screw, which did not participate in the training of ANNs, differed from the nature of the work of the other screws, for which these forces were physically measured using washer sensors. Therefore, an attempt was made to determine other parameters from the signals that could improve the generalization abilities of ANNs. The pattern database was extended to include data related to the time and amplitude of the occurrence of subsequent maxima in the response signals (Figure 7a). These arrival times were expressed as the distance between the maximum excitation amplitude and the local extremes of the response amplitudes. Their values were determined for all signals and analyzed connections. To illustrate the nature of the changes in arrival times, the value $t_{0}$ obtained without a load was subtracted from each of them ($\Delta t=t_{i}-t_{0}$, where i={1,2,…,7}). An example of the obtained time variations in the case of the P1 connection is shown in Figure 7b. Examples in the literature indicate that the relationship between ToF changes and load is most often almost linear [18,19]. In this particular case, with the type of sensors used and the established excitation signal parameters, these relationships are non-linear.

Moreover, although this type of graph does not indicate this, along with the increase in the axial force, the determined arrival times of the individual peaks in the signal were generally constantly reduced. It was expected that the tensioning of the connection would cause screw elongation and a longer wave path would increase the related propagation time. Although there are examples in the literature where the impact of stress on the propagation speed was analyzed [16], a wider discussion of this issue, which is very interesting, is not the main subject of this study.

### 2.5. Artificial Neural Networks

Tasks related to the prediction or identification of parameters are related to the category of so-called inverse problems [12]. Most often, they consist of determining unknown parameters on the basis of values measured experimentally or obtained from numerical simulations. In a situation where knowledge about the observed phenomenon or the degree of its complexity does not allow building an expert system, the most frequently used tools are ANNs.

ANNs are widely used in many areas and tasks. The assumption is that ANNs are able to learn an unknown relationship between input and output data. This typically requires large amounts of data acquired in computational or experimental investigations. In recent years, much attention has been paid to so-called deep learning (DL) [20], and one of the major benefits of using deep models is successful NN learning from fewer training data. In addition, deep learning approaches have proven to be suitable for big data analysis, and hierarchical learning systems show superior performance in several engineering applications [21]. In the approach described in this article, two applications are demonstrated: DL multi-layer perceptron (MLP) was used for signal compression, and DL regression ANNs were trained to predict the axial forces in the screws of the investigated connections.

The learning process consisted of minimizing the computed error value between the target and the network outputs obtained for successive iterations. Testing and validation were carried out based on data that the network had never seen before. The ability to produce such a prediction for the training set is called network generalization [10]. In this article, the mean squared error (MSE) and standard deviation were used as measures of the error obtained at the network output.

The force identification provides information about the predicted value of that force with respect to parameters that are sensitive enough to its changes. The correct selection of these parameters is the most important issue in any identification task. Then, the accuracy of the neural predictor may be obtained by tuning the architecture or different training strategies. For the aforementioned task, feed forward ANNs are commonly used [10]. They consist of an input (first) layer, (usually) a few hidden layers, and an output layer. The number of elements in the input and output layers is determined by the size of the training datasets.

As an alternative to the designated wave parameters (PCA, time and amplitude (TA)), an MLP was trained to reproduce the inputs in the output layer. This kind of network is called an autoencoder. The network is trained so that the signal feed at the input is reproduced at the output, so the input layer and the output layer have the same dimensions. In the middle of the network, there is a hidden layer—called the bottleneck layer—that has fewer neurons than the input layer. This auto-associative neural network is used to compress signal data. After the learning process, the output and hidden layers after the bottleneck are neglected, and only the input, hidden, and bottleneck layers are used. This part of the network is called the encoder, which is used to reduce the dimension of the measured signals. It can be assumed that the parameters obtained from the encoder represent all the information contained in the signals. Moreover, this approach allowed us to perform compression on the input data and to reduce the data dimensionality. In other words, the encoder extracts the most important components of the signal and ignores the less important parts. The encoder, with only one hidden layer (the bottleneck), can be compared by computing the PCA of the input data, while the additional hidden layers introduce some non-linear transformation of the signal [22], and the encoder performs the PCA on a non-linear version of the signals. In this work, various encoder architectures were trained: inl−850−250−bl, where inl={8192,4096,3072} is related to the number of data taken from the measured signal (starting from its beginning) and bl={24,12,6,4}. It was found that the most suitable architecture for the DNN for the compression task was 4096−850−250−12−250−850−4096, and the DNN for the regression task was 13−50−50−50−1.

## 3. Results and Discussion

### 3.1. Force Prediction in Single Connections

The first task was to identify the forces in the bolts in a single connection. Each contained a screw, which was not equipped with a force sensor, but the elastic wave signals were recorded there during a static tensile test. With reference to this particular screw, the axial forces were predicted.

In previous works [11,17], it was assumed that data from all bolts (2, 4, 5) were to be used for the purpose of ANN training, assuming a constant distribution of patterns for testing and validation. The new approach proposed herein involves the learning, testing, and validation patterns being separated into individual bolts from a given connection. Among the possible combinations, the following division of patterns was adopted (short names are provided in brackets; they are also used in the data descriptions in the charts and tables):

- S4 for learning (learn);
- S2 for testing (test);
- S5 for validation (valid);
- S3 for prediction (predict).

This is to achieve the repeatability of force identification for various bolts in the connection and then possibly extend the database to include bolts from the other connections.

After the first series of simulations, it turned out that none of the signal parameter sets (PCA, TA, encoder) allowed us to obtain a satisfactory level of identification accuracy for the axial forces in the screws. This was due to the fact that there were relatively significant differences between the signals measured in the individual bolts. Of course, this can be influenced not only by the excitation pattern and its parameters (especially the frequency), but also by the likely differences in the attachment of the screws (some of them may touch the edge of the hole, while others may not). However, these are limitations arising both in the measurement capabilities and factors that occur in real constructions. For this reason, the obtained pattern database derived from laboratory experiments of connection models with real dimensions is a good test of the proper operation of the developed diagnostic system.

At the current stage of research, it was decided to extend the database by the elongation measured on one of the screws. This measurement was made using inductive sensors applied to both ends of the S1 screw. In this way, the input data gained an additional physical sense, which seems to be the key issue in this case.

The input vectors consisted of the elongation of S1 and:

- Twelve principal components (PCA);
- Six amplitudes of the response signals and theirs six arrival times (TA);
- Twelve parameters obtained from the encoder.

**Figure 8:**
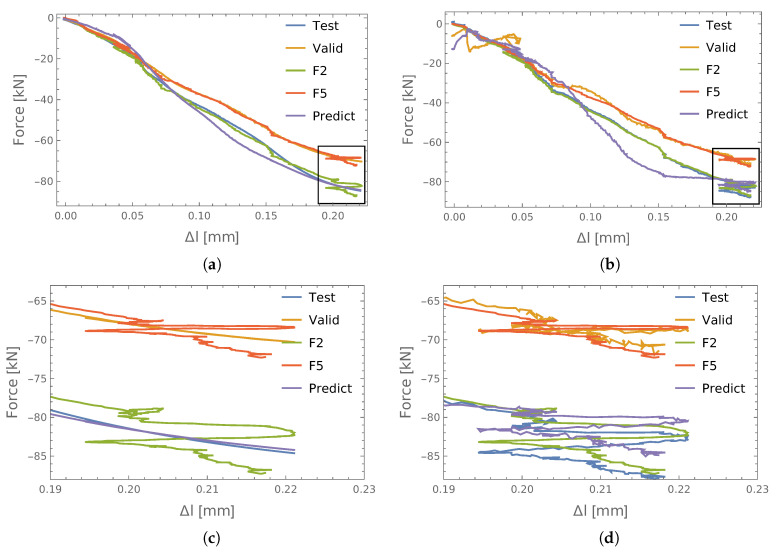
The results of identification forces: P1. (**a**) Encoder; (**b**) PCA; (**c**) details (**a**); (**d**) details of (**b**).

The obtained values of the identification errors for two exemplary connections (P1) with respect to the input data used are shown in Table 2. It can be seen that the smallest learning errors were obtained for the PCA, but this also led to the largest validation errors. Time and amplitude (TA) had the lowest validation error, but reasonably good accuracy was obtained in the case of the encoder. A graphic comparison of the results for the encoder and PCA is shown in Figure 8. Therein, the F2 and F5 forces measured in the S2 and S5 bolts, as well as the respective force values obtained from the ANN (test and valid) are shown. It can be seen there that they are in good agreement, especially the input vector taken from the encoder. In addition, the predicted values of the axial forces in bolt S3 (predict) show the nature of the changes to be very similar to the other screws.

If we look at the fragment of results that are enlarged (Figure 8c,d), we see that training the ANN using the input data taken from the encoder led to the averaging of the identified quantities (Figure 8c), which is very interesting. However, in the case of the principal components (Figure 8d), the results obtained followed the measured values, which in the case of the measurement errors may lead to them becoming stronger.

The same approach was also repeated for the other connections. For each case, it was possible to identify the axial forces in the bolts at a similar level of accuracy. As an example, the list of errors obtained for the P3 connection can be analyzed (Table 3). This time, the best validation results were obtained for the encoder data. Although they were very similar to the TA results, the errors for learning and testing were at a lower level. Therefore, in other cases presented in the paper, encoder data were used as the input to the ANN.

### 3.2. Force Prediction in Sets of Connections

In the next task, an attempt was made to combine the data from two and more connections. The idea was to check whether it was possible to train the diagnostic system on one connection and use it to assess the forces in the bolts of the other connections. Therefore, connections with the same bolt lengths were grouped, and the results of the simulations carried out are presented in the following sections.

#### 3.2.1. P2P3

The first set of connections consisted of two connections (P2P3). They corresponded in terms of the dimensions of the end plates and the class of screws. In this case, two sets of input vectors were also analyzed:

1. The ANN used data from the P2 = {S2, S4, S5} connection to learn, while data from P3 = {S4, S5} were used for testing and P3 = {S2} for validation;
2. The ANN used data from the P3 = {S2, S4, S5} connection to learn, while data from P2 = {S4, S5} were used for testing and P2 = {S2} for validation.

**Figure 9:**
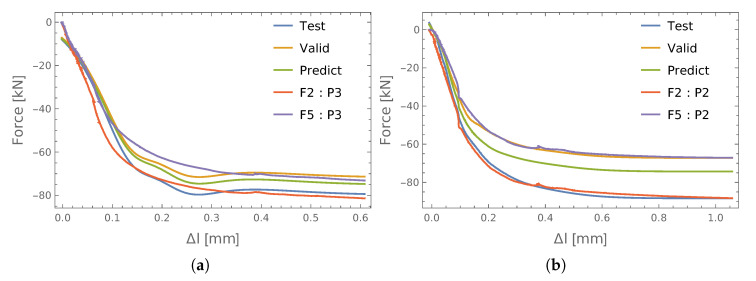
The results of force identification: (**a**) P2 learning, P3 testing; (**b**) P3 learning, P2 testing.

In both cases, the prediction was made with respect to the S3 screws from both connections (P2, P3). The obtained results of testing, validation, and prediction are shown in Figure 9. There was a good agreement between the measured axial forces (F2, F5) and the values obtained from the ANNs.

It can be seen in Table 4 that lower statistical parameters were observed when data from P3 were used for learning and P2 for training and validation.

#### 3.2.2. Set of P2P3P4 Connections

**Figure 10:**
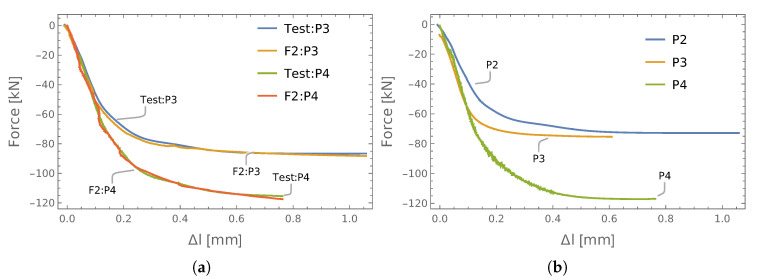
The results of force identification: (**a**) testing; (**b**) prediction.

The promising results obtained for the first set prompted us to extend it with data related to the P4 connection, which differed not only in the dimensions of the front plate, but also in the class of screws. Training patterns in this case were separated as follows: learning was performed on patterns from P2 and P3 connections (i.e., P2 = {S2, S4, S5}, P3 = {S4, S5}), while testing and validation involved a set of patterns related to P3 and P4 connections (i.e., [P3 = {S2}, P4 = {S2}], and P4 = {S4, S5}, respectively). An example of the results obtained from testing and prediction is shown in Figure 10. As before, there is good agreement between the measured axial forces F2 and the values estimated by the ANN (Figure 10a). The predicted values of the forces in the S3 screws (which were not equipped with force sensors) for all three connections are shown in Figure 10b. The nature of their changes is similar to the other bolts in the analyzed connections.

#### 3.2.3. Set of P2P3P4P6 Connections

**Figure 11:**
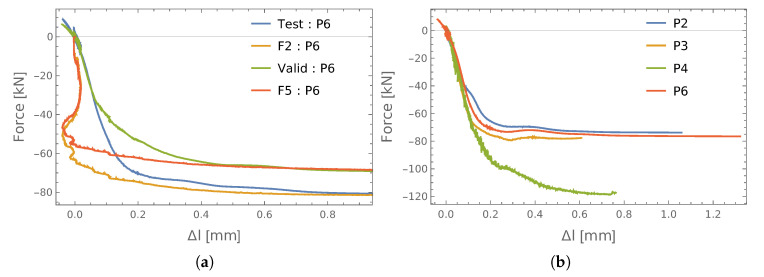
The results of force identification: (**a**) testing; (**b**) prediction.

The dataset used to train the ANN was expanded to include results from the next connection (P6). The patterns in this process were divided according to the following scheme: the learning was carried out on data from bolts P2 = {S2, S4, S5}, P3 = {S4, S5}, P4 = {S4, S5}; the testing involved data related to [P3 = {S2}, P4 = {S2}, P6 = {S2}]; while the validation was performed using data from P6 = {S4, S5}. The obtained results from the testing and prediction of the axial forces in the S2 bolts in the analyzed connections are shown in Figure 11.

The characteristic pattern in this case was the noticeable smoothing of the test results. The determined elongation values indicated that in a certain phase of the tensile test, the length of the S1 screw was temporarily shortened. The real reason, however, may be the fact that the end plates of the P6 connection were deformed and the screws were bent (see Figure 2). Thus, the changes registered by the sensors were the result of the bolt end rotating and not its actual shortening.

Summarizing the results obtained so far, it can be stated that adding information related to the elongation of one of the bolts allows for good estimation of the identified values of the axial forces in the bolts of flange connections. However, taking into account that the application of this approach in practice is very difficult or even impossible, an attempt was also made to replace the bolt elongation with its increments. This idea and preliminary results are briefly described in the next section.

### 3.3. Force Prediction Using Load/Elongation Increments

The concept of identifying forces in bolts using load or elongation increments consists of the fact that the load change in the tested connection is introduced. This is accompanied by the simultaneous measurement of elastic wave propagation and the elongation in one of the screws. Then, the obtained elongation increment is added to the other parameters provided in the input vector to the ANN.

To show the potential of the proposed approach, preliminary simulations were performed on the data related to the P3 connection. For this purpose, the database was divided into relatively small increments. It turned out that better identification results were obtained when these increments were not uniform, but variable with a random Gaussian distribution. Thus, the database used for ANN training consisted of elongation increments of the S1 bolt and the parameters of signals (taken from the encoder) measured at the following bolts: S4 for learning; S2 for testing; S5 for validation; and S3 for prediction.

**Figure 12:**
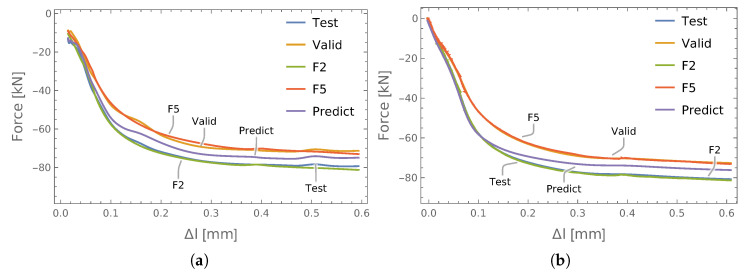
The results of force identification in the P3 connection based on: (**a**) load/elongation increments; (**b**) direct elongation of the bolt S1.

The results of the preliminary tests are presented in Figure 12a together with the results corresponding to the ANN trained directly using the elongation of the S1 screw (Figure 12b). It can be seen that there were some differences in the accuracy of testing, validation, and prediction of the axial forces. The error values calculated on this basis are collected in Table 5. Despite the clearly superior results in the case of training the ANN with the direct elongation of the S1 screw, the results of both approaches remained in good agreement with the results of the experimental measurements of the F2 and F5 axial forces in the S2 and S5 screws. Therefore, in future studies, this approach will also be extended to other connections.

**Figure 13:**
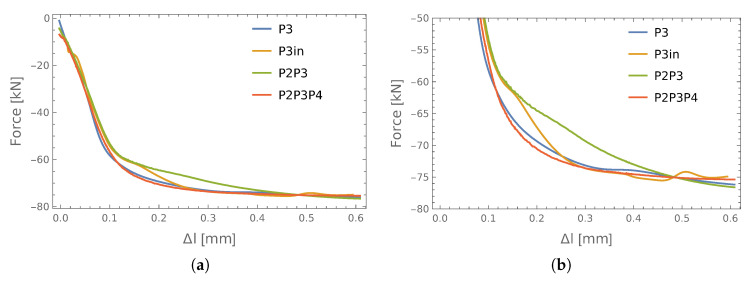
The result of the prediction forces with respect to the P3 connection: (**a**) comparison of the studied input vector scenarios; (**b**) details of (**a**).

As a summary of the approaches analyzed so far, the results of axial force prediction in the S3 bolt of the P3 connection (which was not equipped with a force sensor) are summarized in Figure 13a. There, we can see the results obtained for the ANNs trained on the basis of data from a single connection (P3 with elongation, P3inwith elongation increments), two connections (P2P3), and three connections (P2P3P4). The results related to P3 and P2P3P4 are similar to each other (which is clearly seen in Figure 13b), while P3in and P2P3 slightly differ from them.

## 4. Conclusions

One of the great advantages of the research presented herein is that it is based on experimental measurements carried out on a set of laboratory models of real flange connections. The main assumption of the proposed approach was that elastic waves carry information about the axial force in the bolt in which they propagate. Due to the significant differences in the measured signals, their compression using the MLP encoder was of great importance. By adding the variable of the elongation of one screw to the input vector, it became possible not only to achieve satisfactory identification results, but to achieve generalization capabilities. Moreover, this was not only possible with respect to one single connection, but after merging the signal parameters, it was possible for several investigated connections. Therefore, it can be concluded that the use of the MLP encoder (signal compression) and the addition of a bolt elongation (a physical variable) made it possible to find the relationship (ANN trained) between the propagating elastic wave and the axial force in the bolt subjected to tension.

On the basis of the trained ANN, axial force prediction was carried out for bolts that were not equipped with force sensors. The values obtained were consistent with those of other bolts, which was not possible in previous studies [11,17]. In this work, the dimensionality reduction of measured wave signals (neural network encoder) gave meaningful improvement to the results of the identification of the force in the bolts. Moreover, this new approach enabled: (1) the prediction of forces in bolts where they were not directly measured; (2) simultaneous analysis of data related to several connections. Obtaining the expected values of the prediction of axial forces was completely impossible in the previously considered stage of the research due to a serious problem with achieving the generalizing ability of ANNs [11,17]. The approach presented in this paper has eliminated this problem.

The article also presents the preliminary results of the identification of the axial forces, in which instead of the elongation of a single screw, elongation increments were used. Thanks to this, the proposed approach also has the potential for practical applications in real non-destructive test (NDT) measurements. Therefore, in future studies, this idea will also be extended to other connections. It will also be a good opportunity to include, in addition to processing elastic wave signals, the filtering of data related to the measurement of axial forces in bolts, which also influence the prediction accuracy.

Furthermore, it is worth mentioning that during the static tensile test, the force applied by the testing machine was also recorded. Therefore, it is also possible to use this in the pattern database information regarding load increments, which, in practice, may be more useful than measurements of elongation changes.

## Figures and Tables

**Table 1:** Dimensions of the investigated flange connections.

Connection	Symbol	Plate Geometry ($t_{f}$/$e_{1}$/$e_{2}$/D) (mm)	Screws	
			Class	Length (mm)
P1	BK25.6.2	25/35/25/190	6M16 8.8	125
P2	BK 25.6.1	25/35/25/190	6M16 5.8	85
P3	BT25.6.1	25/35/25/190	6M16 5.8	85
P4	BK 15.6.4	15/45/25/210	6M16 8.8	85
P5 *	BK 15.6.3	15/45/25/210	6M16 5.8	85
P6	BT 10.6.1	10/35/25/190	6M16 5.8	85

* data related to this connection were not used – details are given in the text.

**Table 2:** Comparison of the learning, testing, and validation errors: P1.

Method	Learn		Test		Valid	
	MSE $\cdot 10^{5}$	$\sigma \cdot 10^{3}$	MSE $\cdot 10^{5}$	$\sigma \cdot 10^{3}$	MSE $\cdot 10^{5}$	$\sigma \cdot 10^{3}$
PCA	**14.6**	**12.1**	41.1	20.3	134.9	36.7
TA	139.4	37.3	164.3	35.2	**13.2**	**11.5**
Encoder	27.5	16.6	**32.7**	**18.1**	16.6	12.9

**Table 3:** Comparison of the learning, testing, and validation errors: P3.

Method	Learn		Test		Valid	
	MSE $\cdot 10^{5}$	$\sigma \cdot 10^{3}$	MSE $\cdot 10^{5}$	$\sigma \cdot 10^{3}$	MSE $\cdot 10^{5}$	$\sigma \cdot 10^{3}$
PCA	**2.75**	**5.24**	4.24	7.23	36.2	19.1
TA	34.1	18.5	39.5	19.8	5.98	7.73
Encoder	3.83	6.20	**4.03**	**3.55**	**3.78**	**6.11**

**Table 4:** Comparison of the learning, testing, and validation errors: P2P3.

Method	Learn		Test		Valid	
	MSE $\cdot 10^{5}$	$\sigma \cdot 10^{3}$	MSE $\cdot 10^{5}$	$\sigma \cdot 10^{3}$	MSE $\cdot 10^{5}$	$\sigma \cdot 10^{3}$
P2-learn, P3-test and valid	41.6	24.2	287.4	53.6	290.1	53.8
P3-learn, P2-test and valid	39.8	19.9	54.3	23.3	30.0	17.3

**Table 5:** Comparison of the learning, testing, and validation errors: P3 and P3 increments.

Method	Learn		Test		Valid	
	MSE $\cdot 10^{5}$	$\sigma \cdot 10^{3}$	MSE $\cdot 10^{5}$	$\sigma \cdot 10^{3}$	MSE $\cdot 10^{5}$	$\sigma \cdot 10^{3}$
P3	3.83	6.20	4.03	3.55	3.78	6.11
P3 increments	18.8	13.7	26.8	16.4	29.7	17.2

## Abbreviations

The following abbreviations are used in this manuscript:

ANN	Artificial neural networks
P*j*	Connection number, where j={1,2,…,6}
DL	Deep learning
F*i*	Force magnitude in the S*i* screw, where i={1,2,…,6}
DNN	Deep neural network
MLP	Multi-layer perceptron
MSE	Mean squared error
NDT	Non-destructive test
PCA	Principal components analysis
PZT	Piezoelectric transducer
S*i*	Screw number, where i={1,2,…,6}
SHM	Structural health monitoring
SNR	Signal-to-noise ratio
TA	Arrival time and amplitude
ToF	Time of flight
bl	A bottleneck layer of the neural network
inl	An input layer of the neural network
σ	Standard deviation
